# Multi-Method Complex Approach for Hydration Assessment Does Not Detect a Hydration Difference in Hemodialysis versus Peritoneal Dialysis Patient

**DOI:** 10.3390/diagnostics10100767

**Published:** 2020-09-29

**Authors:** Anna Adamska-Wełnicka, Marcin Wełnicki, Paweł Krzesiński, Stanisław Niemczyk, Arkadiusz Lubas

**Affiliations:** 1 Clinic of Nephrology and Internal Medicine, Centre of Postgraduate Medical Education, 01-813 Warsaw, Poland; 2 3rd Clinic of Internal Medicine and Cardiology, Medical University of Warsaw, 02-091 Warsaw, Poland; mwelnicki@wum.edu.pl; 3 Clinic of Cardiology and Internal Medicine, Military Institute of Medicine, 04-141 Warsaw, Poland; pkrzesinski@wim.mil.pl; 4 Department of Internal Medicine, Nephrology and Dialysotherapy, Military Institute of Medicine, 04-141 Warsaw, Poland; sniemczyk@wim.mil.pl (S.N.); alubas@wim.mil.pl (A.L.)

**Keywords:** dialysis, lung ultrasound, B-line score, electric bioimpedance, impedance cardiography

## Abstract

Assessment of hydration status is essential in monitoring the effectiveness of renal replacement therapy and is usually based on physical examination. However, comparisons of hydration status achieved with different dialysis methods are not conclusive. We compared the hydration status of patients on chronic hemodialysis (HD, *n* = 60) and peritoneal dialysis (PD, *n* = 20) in a comprehensive assessment including physical examination and additional methods. The mean age of the 80 chronically dialyzed patients (53 males, 27 females) was 58.1 ± 13.9 years. The clinical evaluation took into account the presence of peripheral edema, dyspnea, and crackling over the lung fields. Additional tests included lung ultrasound, electrical bioimpedance (performed in 79 patients), impedance cardiography, ultrasound assessment of large abdominal vessels (performed in 79 patients), select echocardiographic parameters (obtained in 78 patients), and serum NT-proBNP concentration. Residual diuresis volume was significantly higher in the PD group. We found no significant differences between the two groups in any other baseline characteristics or in the results of the clinical examination or additional tests. The use of different methods for assessing hydration does not allow differentiation of patients treated with dialysis in terms of the dialysis technique used. Therefore, it seems reasonable to use common algorithms to objectify the hydration status of these patients.

## 1. Introduction

End-stage renal disease is characterized by a number of abnormalities that disrupt the body’s homeostasis. Overhydration is a particularly important problem, especially in patients on dialysis. In less advanced stages of chronic kidney disease (CKD), overhydration accelerates the loss of renal function and the time to start renal replacement therapy [1]. Overhydration also increases arterial stiffness and left ventricular hypertrophy, leading to the development of hypertension and heart failure [2]. In addition, the condition is associated with an increase in total and cardiovascular mortality in both hemodialysis (HD) and peritoneal dialysis (PD) patients [3,4,5,6]. Therefore, the clinical consequences of overhydration justify considering water as a uremic toxin [7]. On the other hand, hypovolemia is a risk factor for increased mortality [8]. Hypovolemic episodes are one of the basic mechanisms responsible for the decrease in residual renal function in HD patients [9,10] Therefore, optimizing hydration status remains a key therapeutic goal in nephrology patients.

Achieving and maintaining proper hydration in a dialysis patient is a serious challenge. Decisions on the volume of ultrafiltration are made during HD, or after prior analysis of weight gain between HD procedures. Significant interdialytic weight gain, defined as a weight gain > 4.8%, increases the overall mortality of HD patients [11]. However, the risk of death associated with high interdialytic weight gain is much lower than the risk associated with chronic overhydration [12]. Patients without signs of hypovolemia or advanced hypervolemia after uncomplicated HD can reach their target body mass (“dry mass”). Dry mass is assumed to correspond to the optimal hydration status [13]. However, studies have shown that approximately 20% of patients do not achieve this therapeutic goal [14].

According to some researchers, overhydration occurs with a similar frequency in HD and PD patients and is associated with negative clinical consequences in both groups [15]. However, most data on the occurrence and clinical significance of overhydration concern HD patients. Reports on PD patients are scarce and inconclusive. For example, in the European Body Composition Monitoring (EuroBCM) study concerning patients with PD, severe overhydration was found in only 25.2% of patients based on bioelectrical impedance analysis (BIA) [16]. However, the results of the EuroBCM study do not correspond to the prevailing opinion that PD promotes overhydration. Studies comparing the hydration status of patients based on the method of dialysis are few, especially those taking into account diagnostic methods other than clinical assessment and body composition monitoring (BCM).

Accurately assessing the hydration status of dialysis patients is one of the main problems. Conclusions based on symptoms and signs, such as high arterial pressure, shortness of breath, auscultatory changes over the lung fields, peripheral edema, jugular vein widening, hepatomegaly, or weight gain are characterized by low sensitivity and specificity, though it remains the basic diagnostic tool [2,17]. Diagnostic tools that can clarify the data on the patient’s hydration status obtained from the clinical assessment are still needed. The ideal method should be accurate and repeatable, with simple and quick implementation, and potential for bedside use. Additional tests that we usually use to assess the state of hydration are X-ray, electrical bioimpedance, assessment of the concentration of natriuretic peptides (brain natriuretic peptide (BNP) and N-terminal pro-brain natriuretic peptide (NT-proBNP)), impedance cardiography (ICG) and other methods of segmental bioimpedance, and ultrasound methods (inferior vena cava (IVC) assessment, echocardiography, and the increasingly popular lung ultrasound (LUS)). Notably, none of the tests determine the state of hydration in an ideal way, and the overall assessment may be the conclusions drawn from results obtained with each of the methods.

The aim of the present study was to compare the results of a comprehensive hydration assessment of chronically dialyzed HD and PD patients carried out by clinical assessment and select additional methods.

## 2. Materials and Methods

### 2.1. Patients

A total of 80 chronically dialyzed patients (53 males, 27 females; mean age 58.1 ± 13.9 years) remaining in the care of one dialysis center were included in the study between May 16 and 8 November 2018. HD (*n* = 60) patients were treated three times a week, and hydration status (clinical assessment and additional tests as described below) was assessed immediately prior to central HD. In PD patients (*n* = 20; 2 on continuous cyclic and 18 on continuous ambulatory peritoneal dialysis), hydration status was assessed during the patient’s routine visit to the lead center. The criteria for study inclusion and exclusion are presented in Table 1. Each patient was examined only once. The study was conducted in accordance with the Declaration of Helsinki. The presented analysis is part of a research project that has received a positive opinion from the Bioethics Committee operating at the Military Medical Institute in Warsaw (resolution number 93/WIM/2018 21/03/2018). The study was carried out as part of the statutory project: grant for young scientist No. 537. All patients provided written consent to participate in the study.

### 2.2. Clinical Assessment

In the clinical assessment, attention was paid to the presence and severity of lower limb edema (four-grade scale), dyspnea declared by the patient (New York Heart Association, NYHA scale), and the presence of crackling detected during lung auscultation (scale adapted from the LUST study [18]). The clinical evaluation scheme is shown in Table 2. As part of the clinical evaluation, basic anthropometric measurements, allowing body mass index (BMI) and body surface area (BSA) to be calculated, were also taken, and pulse rate and blood pressure were estimated.

Based on the clinical evaluation, a dichotomous division was adopted. Patient without any symptoms and signs were classified as non-hypervolemic, others as hypervolemic. The assessment of hydration status was objectified using ultrasound (LUS, IVC, and echocardiography), impedance (electrical bioimpedance and ICG), and laboratory values (NT-proBNP concentration).

### 2.3. Ultrasound Investigation

Ultrasound examinations were carried out using the Lumify Philips porTable 1–4 MHz sector probe using dedicated presets for lung assessment in 2D and cardiac examination in 2D and M-mode options. LUS was performed with the patient lying supine. To assess lung congestion, a protocol of 28 scans (probe touchdowns) was used to calculate the B-line score parameter (Table 3 and Table 4).

The ultrasound assessment of the IVC was performed on the patient in a supine position with visualization in the sub-sternum projection. The maximum dimension of the IVC was indexed to BSA, obtaining the parameter referred to as the indexed diastolic dimension of the IVC (IVCDi) (Table 5). In addition, the collapsibility of the IVC (IVCCi) was calculated using the following formula.

([Exhalation IVC diameter − inhalation IVC diameter]/Exhalation IVC diameter) × 100%(1)

Echocardiography was performed according to current standards [23]. Measurements were recorded in the parasternal long-axis view. Using the capabilities of the portable ultrasound probe software, we evaluated the following parameters; interventricular septum thickness at end-diastole, left ventricular end-diastolic dimension, and posterior wall thickness at end-diastole. Based on the data, left ventricular muscle mass was calculated according to the American Society of Echocardiography convention. The left ventricular muscle mass score was also indexed to the patient’s body surface area (left ventricular mass indexed to body surface area (LVMI)).

### 2.4. Impedance Methods

Total body electrical bioimpedance testing was performed with the Body Composition Monitor (Fresenius Medical Care, Germany, software version 3.3.x). The measurement procedure was carried out in accordance with the principles of device use. The patient was in a supine position for approximately 5–10 min before starting the examination. During the examination, the patient’s arms did not touch their body, and the lower limbs were not joined. Metal objects and devices had been removed from the surroundings so that they did not come into contact with the patient. Electrodes for examination were placed on the arm and foot of the right or left side of the body; in the case of arterio-venous dialysis fistula, the opposite side was chosen. Two electrodes were placed on the dorsal surface of the hand above the metacarpal joints and along the wrist joint cut-off line and two more above the metatarsophalangeal joints and on the foot joint cut-off line. During the examination, the patient did not move or talk. In the assessment of overhydration, the BCM-OH (overhydration according to Body Composition Monitor) parameter was used to express excess or deficiency of water in liters. It is assumed that the value of BCM-OH should be between −1.1 to 1.1 L (if BCM-OH is less than −1.1 L it indicates hypovolemia, if it is more than 1.1 L, it indicates hypervolemia)

Impedance cardiography, a noninvasive method of hemodynamic assessment [24], was performed using a Niccomo™ device (Medis, Ilmenau, Germany). During the examination, the patient remained in a supine position and was forbidden from moving and talking, and objects and metal devices were removed from the environment. Symmetrically, eight electrodes were arranged on both sides of the patient’s neck and in the middle axillary line within the chest. Four power supply electrodes, so-called “external sensors” were located on the neck above and on the chest below the four voltage electrodes. Voltage electrodes received changes in the potential in the electric field between the external sensors (impedance cardiogram curve) of the chest area, including the heart and large vessels. In addition, three electrodes recording the electrocardiogram were placed on the anterior chest surface. The device performed cyclic (every 2 min) blood pressure measurements using a sphygmomanometer cuff placed on the patient’s arm. The hemodynamic parameters were recorded for a minimum of 10 min and after a period of at least 1-min stabilization of the recording quality. Due to the lack of recommended norm in dialyzed patients for impedance cardiography parameters, the indexed parameter was used (thoracic fluid content/height, TFC/h).

### 2.5. Laboratory Tests

NT-proBNP concentrations were determined in serum from venous blood using the electrochemiluminescence immunoassay (ECLIA) method using a Roche COBAS INTEGRA 400 plus analyzer. Reference values were 0–194 pg/mL.

### 2.6. Statistical Analysis

Data analysis was carried out using STATISTICA version 12.0 (StatSoft Inc., Tulsa, OK, USA). For quantitative variables, the average values with standard deviations or the median with minimum and maximum values are presented. For qualitative variables, the number and frequency of mode are given, with their number and percentage depending on the type of data presented. The distribution of values for quantitative variables was checked using the Shapiro–Wilk test. For quantitative variables with a normal-like distribution, the differences in groups were tested using the Student’s t-test for unrelated variables and the correlations using the Pearson correlation coefficient. For unrelated quantitative variables with a non-normal distribution, the differences were tested using the Mann–Whitney U test and the correlations using the Spearman correlation coefficient. Significant differences between qualitative variables were assessed using the chi-squared test. To check a concordance between clinical assessment and other methods a Kappa test of Cohen was also performed. Data loss was not replaced by the mean but was omitted. The result of the statistical test was considered significant if the *p*-value was less than the type I error (α = 0.05).

## 3. Results

The baseline characteristics of the study groups are presented in Table 6. One HD patient was not assessed by BCM due to the presence of dressings on the lower limbs (diabetic foot). Due to poor imaging conditions in two PD patients, no echocardiographic measurements were taken and one of these patients also did not have results for IVCDi. The groups did not differ significantly in regards to age, time of dialysis, blood pressure, heart rate, BMI, or body surface. In contrast, patients from the PD group had significantly greater residual diuresis (urine output) compared to the HD group (*p* = 0.006; Table 6). The study groups did not differ in terms of hypertension, diabetes, coronary artery disease, heart failure, or cerebrovascular disease.

The results of the clinical assessment of patients from both groups in the context of signs and symptoms of overhydration are presented in Table 6 and Table 7. We found no significant differences in the severity of dyspnea, edema, or the presence of crackles between HD and PD patients. There were no significant differences in numbers of hypovolemic, euvolemic, and hypervolemic patients in both groups (Table 8). In two PD patients with no symptoms and signs of hypervolemia in clinical evaluation neither had results of additional studies indicating hypovolemia. In 15 HD patients with no symptoms and signs of hypervolemia in clinical evaluation none had an IVCDi value or BCM-OH value indicating hypovolemia. Kappa test of Cohen was performed to compare the ability of each method to correctly characterize the hydration status of the patients (Table 9). Each additional examination method had a better concordance (fair to moderate agreement) with a specific kind of clinical assessment than with overall clinical evaluation (slight to fair agreement) of hydration status.

We also found no significant differences between the HD and PD groups in terms of hydration status based on the results of additional tests (Table 10).

## 4. Discussion

To the best of our knowledge, this study is the first to compare the hydration status of PD and HD patients using a number of complementary diagnostic methods: clinical examination; assessment of fluid content in the chest by two methods (LUS and ICG); and intravascular volemia including myocardial overload (ECHO), IVCDi, IVCCi, NT-pro-BNP, and BCM. This is the basis for saying that PD patients do not differ significantly from HD patients in terms of hydration status in both clinical assessment and objective results of additional tests. Furthermore, the additional content of dialysis fluid in the peritoneal cavity and significantly higher daily diuresis in the PD group did not affect the clinical significance of the presented results.

The ability to control hydration in HD and PD patients varies significantly and may influence clinical decisions. The planning of dehydration, i.e., ultrafiltration immediately before each dialysis procedure, and the possibility of changing this parameter during its duration allows the removal of excess water accumulated between treatments and quick achievement of the controlled target weight within a few hours. In contrast, in PD patients, the volume of ultrafiltration depends on hydration status, peritoneal properties, and the type of dialysis fluid. Intervention efficiency can be achieved more slowly in PD patients than HD patients. In addition, PD patients report for medical check-ups every 2–6 weeks, which is an advantage of this method, but also increases the risk of worse control of hydration status. The presence of fluid in the peritoneal cavity in PD is due to the dialysis method, but can also affect the results of additional tests assessing overhydration.

Taking all of the above into consideration, there is a widespread belief that PD patients may be expected to have worse hydration status and a higher frequency of overhydration than HD patients. The research results available in the medical literature regarding this issue are ambiguous. Plum et al. compared BIA parameters and atrial natriuretic peptide levels obtained in 39 HD and 43 PD patients, and found that PD patients have higher total body water (TBW) and extracellular body fluid (ECF) values assessed by BIA compared to HD patients, both before and after dialysis [25]. However, in the discussion, the authors emphasized that the assessment of extracellular compartments in BIA does not allow differentiation of intravascular and extravascular volumes. They intended to resolve these doubts by examining the concentration of ANP as a reflection of the increase in intravascular volume, but found comparable average values in both groups. The results of the hydration assessment in the present study turned out to be more consistent; PD and HD patients did not differ in NT-proBNP concentration or BCM-OH results. However, the use of other natriuretic peptides (ANP vs. NT-proBNP) and bioimpedance parameters (TBW, ECF vs. BCM-OH estimation) may be relevant. Moreover, in the Plum et al. study, the HD and PD groups did not differ by daily diuresis (<1.0 L), whereas in our study, the mean daily diuresis was significantly higher in the PD group (~1.4 L) than the HD group (~0.7 L). This may explain the better hydration control of PD patients in our study. Aguiar et al. concluded that this method does not have to be associated with chronic overhydration after observing 30 PD patients for 2 years. At the beginning of their observations, BIA indicated significant overhydration in less than 7% of respondents, and this percentage did not change significantly during the 2-year follow-up. At the end of the observation period, not only the BIA results, but also the NT-proBNP concentrations of the examined patients, were comparable [26].

Similar results as in the present study were reported by Oe et al., who did not find significant differences in BIA results or ultrasound assessments of the IVC between PD and HD when comparing the degree of overhydration in much smaller groups (11 PD and 20 HD tested before dialysis) using only two methods [27]. Notably, similar to the present study, PD patients had much higher residual diuresis than HD patients. Yao et al. also used BIA, but assessed a much larger population: 95 PD patients and 75 HD patients before middle HD per week [28]. In this study, the mean BIA-OH values in both groups were comparable. The authors also did not observe significant echocardiographic differences, including LVMI, between the groups. Interestingly, in their conclusion, Yoa et al. supported the statement that PD patients are more overhydrated than HD patients, likely based on the assumption adopted in the regression analysis. The average values of BIA parameters determined before and after HD were used in calculations for HD patients [28]. The aim of our study was to verify clinical assessment before HD; in this regard, our observations are consistent with the results reported by Yao et al. Our results are also consistent with the observations of Papakrivopoulou et al., who compared the hydration status of 72 HD patients and 115 PD patients [29]. PD patients had significantly higher residual diuresis. All parameters assessed by BIA were comparable in both groups if pre-dialysis results were taken into account for HD patients [29]. The mean NT-proBNP concentrations in both groups were also comparable. Finally, Papakrivopoulou et al. did not find significant differences in the echocardiography results. The studied group was characterized as asymptomatic in regards to overhydration, but the ECW/TBW ratio exceeded the normal range in >30% of patients [29]. This finding confirms the need to verify subclinical overhydration using objective results from additional studies.

The use of comprehensive volemic assessment in our study was intended to increase the strength of scientific evidence for a planned comparison of hydration status in both groups of patients. The position of BIA in the assessment of nephrology patients is well established. Moissl et al. compared parameters obtained during bioimpedance of the whole body with the results of determinations made using isotope tests, showing high compliance of these methods [30]. Modification of the ultrafiltration size during HD based on BIA has also been shown to improve the control of hypertension, reduce vascular stiffness, and reduce left ventricular mass [31]. On the other hand, the whole body’s electrical bioimpedance assumes that the human body is a cylinder with uniform conductivity. Thus, the total impedance in the BIA test depends as much as 90% on the impedance of the limbs, which is a frequently raised limitation of this method. Therefore, segmental bioimpedance analysis (SBIA) has been suggested to better reflect hydration status [32]. In an earlier study of 40 HD patients, we showed that the only element of clinical assessment independently affecting the determination of overhydration in the whole body bioimpedance test is the severity of edema (R^2^ = 0.44; *p* <0.0001) [33]. Therefore, BIA likely underestimates overhydration in non-edema patients with fluid redistribution mainly within the chest compartment. This justifies the use in everyday practice of diagnostic tools assessing the water content of the chest, such as LUS and ICG.

The interstitial syndrome found in LUS is an expression of the presence of water in the extravascular lung space (EVLW) [34]. Agricola et al. observed a correlation between the number of B lines and EVLW measured by thermodilution (r = 0.42, *p* = 0.001; PICCO System), EVLW assessment in chest radiology based on the Extravascular Lung Water Score (r = 0.60, *p* = 0.0001), and wedge pressure in pulmonary capillaries (r = 0.48, *p* = 0.01) [34]. A significant relationship between the presence of the B line and pulmonary interstitial edema was confirmed by computed tomography [35]. In many studies, a significant relationship has also been observed between the number of B lines and the declared degree of dyspnea according to NYHA classification in both HD and PD patients [18,36,37,38]. Much evidence possibly confirms lung congestion on LUS in asymptomatic, and even optimally hydrated, patients according to other studies, regardless of the method of dialysis [36,37,38]. The lack of differences in the mean B-line scores in the PD and HD groups in this study is consistent with the results of the clinical assessment.

Impedance cardiography is a noninvasive method for hemodynamically monitoring a patient, allowing estimation of thoracic fluid content, reflecting the amount of both intracellular and extracellular fluid [39]. This indicator is useful both in differentiating causes of dyspnea [40,41,42] and in optimizing diuretic treatment [39,43]. However, the number of studies conducted to date regarding the use of ICG in dialysis patients is limited. In one publication, Wynne et al. observed a reduction in thoracic fluid content during HD. They also found a correlation between thoracic fluid content reduction and ultrafiltration (r = 0.58, *p* < 0.001) [44]. In another study of PD patients, no significant relationship was found in the logistic regression analysis between the assessment of hydration status based on a subjective and physical examination, and BNP and TFC levels [45]. In our group, no significant differences in TFC were observed between HD and PD patients, which is consistent with the results of other studies. This complementarity suggests the possibility of broader use of ICG in nephrology.

Ultrasound assessment of the IVC allowed evaluation of vascular filling [46]. In one study performed on patients prior to HD in dry weight or above, IVC assessment was useful for identifying hypovolemia [47]. The features of reduced intravascular volume, despite seemingly optimal volemia, were demonstrated in 39% of subjects, confirming the individual variability in intravascular and extravascular fluid distribution. In our study, this method also revealed no differences between HD and PD patients. Ultrasound of the IVC has not yet found wider application in assessing the hydration status of patients on PD. Therefore, as in the case of ICG, the results of our study are an important contribution to current knowledge about the potential applications of this method in dialysis patients.

In summary, a comprehensive assessment of hydration status using complementary methods did not show significant differences between HD and PD patients. Our observations regarding BIA results, average NT-proBNP values, and analyzed echocardiographic parameters are consistent with the observations of other authors. In a recently published review of studies on hydration status in PD patients, Alexandrou et al. point to BIA and LUS as potentially complementary methods [48]. The usefulness of BIA in assessing the hydration status of PD patients was analyzed in studies on groups of patients larger than ours. Though, to the best of knowledge, comparable results from LUS, ICG, and IVC ultrasound in HD and PD patients are unique. Compliance of the LUS, ICG, and IVC ultrasound results with the clinical assessment and BIA increases their clinical value and encourages further research using these methods in renal replacement patients. Whether these methods have independent diagnostic value or increase the discriminatory power of hydration assessment algorithms in select patients is unclear.

Despite the promising conclusions, the present study has several limitations. First, the HD group was larger than the PD group, which could affect the results of the comparisons. On the other hand, the results reported by Papakrivopoulou et al. (BIA, ECHO, and NT-proBNP) for the inverse distribution of the study groups (PD > HD) were consistent with ours [25]. Doubts may also be raised by comparing the hydration status of HD patients prior to HD (potentially overhydrated) with PD patients, who remain in a relatively constant euvolemic state. Based on this assumption, the lack of differences between the compared groups suggests a relatively greater overhydration of PD patients, as suggested by other researchers [24]. Therefore, solving the issue of greater overhydration of PD patients compared to HD patients would require supplementing the HD group assessment with post-dialysis measurements. In the end, in our study, groups did not differ in the quantity of overhydrated patients. However, this lack of differences in the results of additional methods could be related to the rather small sample size of PD group and should be interpreted with caution. On the other hand, based on our results, to show a significant difference in BCM-OH between PD and HD groups, with the power of the test of 80%, the estimated sample size should overcome 455 patients. Nevertheless, presented sample size of PD group relates to the one dialysis center (mean 22.5 patients/center) [16]. Thus, our results, showing no differences between PD and HD patients, appear to be adequate in everyday clinical practice. In conclusion, the use of different methods of assessing hydration status does not differentiate dialysis patients in terms of the dialysis technique used. In the absence of differences in the hydration status of patients on chronic HD and PD in both the clinical assessment and the results of additional methods, it seems reasonable to use common algorithms to objectify the results of clinical assessment of hydration status.

## Figures and Tables

**Table 1:** Criteria for inclusion and exclusion in the study.

Inclusion criteria	Informed consent to participate in the study Chronic renal replacement therapy by hemodialysis or peritoneal dialysis for end-stage renal disease for at least 3 months In HD group, treatment with hemodialysis 3 times a week Age > 18 years of age
Exclusion criteria	Lack of patient’s informed consent to participate in the study Cancer during treatment or up to 2 years after ending the treatment Active, clinically significant inflammation History of dialysis peritonitis during the last 2 months Other severe co-morbidities, except for cardiovascular diseases, with prognosis of short survival, e.g., severe liver failure Known lung disease, e.g., fibrosis, severe obstructive pulmonary disease, pneumothorax, and pulmonary hypertension The amount of fluid in the pleural cavities makes it impossible to assess lung congestion on ultrasound Hemoglobin < 8.0 g/dL Condition after implantation of cardioverter-defibrillator or cardiac pacemaker Significant chest deformity, limb amputation, height < 140 cm or >220 cm; significantly increased body weight (>150 kg) Condition after sternotomy or other surgery within the chest (<24 h, optimal time to perform impedance cardiography approximately 4 weeks after surgery) Known severe aortic and mitral regurgitation Intra-aortic counterpulsation Mental disorders limiting cooperation with patient Increased orthopnea/dyspnea preventing adopting a horizontal position for the time necessary to perform tests Bad quality of ultrasound imaging

HD: hemodialysis patients; PD: peritoneal dialysis.

**Table 2:** Criteria for clinical evaluation of patients.

Edema		Dyspnea According to NYHA		Crackles in the Lung (Adopted from LUST Study)	
**0**	No	**I**	Without limiting physical activity—ordinary physical activity does not cause more fatigue, shortness of breath, or palpitations	**1**	No crackles
**1**	Ankles only	**II**	A slight limitation of physical activity—no complaints at rest, but ordinary activity causes fatigue, palpitations, or shortness of breath	**2**	I am uncertain about the presence of fine crackles
**2**	To calf high	**III**	A significant reduction in physical activity—no complaints at rest, but less than normal activity causes the onset of symptoms	**3**	Definite fine crackles at lung base
**3**	Above the knees	**IV**	Any physical activity causes discomfort, signs of heart failure occur even at rest, and any activity intensifies the discomfort	**4**	Moderate crackles
				**5**	Bilateral, diffuse crackles

NYHA: New York Heart Association. LUST: Lung Water by Ultrasound Guided Treatment in Hemodialysis Patients.

**Table 3:** Scheme of ultrasound examination of the lungs (*n* =28 scans) [18,19,20,21].

Right Lung				IS	Left Lung			
MAL	AAL	MCL	PL		PL	MCL	AAL	MAL
13	9	5	1	II	17	20	23	26
14	10	6	2	III	18	21	24	27
15	11	7	3	IV	19	22	25	28
16	12	8	4	V				

MAL—Middle axillary line; AAL—Anterior axillary line; MCL—Middle clavicular line; PL—Parasternal line; IS—Intercostal space.

**Table 4:** Classification of pulmonary congestion based on lung ultrasound [20].

**Class**	**B-Line Score**	**Pulmonary Congestion**
**0**	<5	No
**1**	≥5 i <15	Mild
**2**	≥15 i <30	Moderate
**3**	≥30	Severe

**Table 5:** Volume state in relation to the measuring ranges of the inferior vena cava diameter index to the body surface and the inferior vena cava collapsibility index [22].

Volume State	IVCDi (mm/m^2^)	IVCCi (%)
**Hypovolemia**	<8	>75
**Euvolemia**	8 ≤ i ≤ 11.5	40 ≤ i ≤ 75
**Hypervolemia**	>11.5	<40

IVCDi: inferior vena cava diameter idexed to body surface area; IVCCi: inferior vena cava collapsibility index.

**Table 6:** Basic characteristics of the study groups.

Measure	HD		PD		*p* Value
	Mean	SD	Mean	SD	
**Age (years)**	58.55	14.30	56.75	13.03	0.457
**Time of dialysis (months)**	36.93	30.48	27.95	24.86	0.192
**Urine output (mL/day) ^i^**	716.67	642.73	1370.00	935.89	0.006
**BSA Dubois (m^2^)**	1.92	0.23	1.90	0.25	0.702
**BMI (kg/m^2^)**	27.74	5.11	28.84	6.52	0.701
**SBP (mmHg)**	139.78	18.28	137.90	26.14	0.723
**DBP (mmHg)**	82.53	12.57	78.95	8.33	0.239
**HR (L/min)**	69.20	9.34	70.70	10.28	0.548
**CRP (ug/dL)**	0.71	1.43	0.85	0.89	0.271
**HGB (g/dL)**	11.09	1.13	11.36	1.78	0.785
**Creatinine (mg/dL)**	8.58	2.63	7.45	2.26	0.126
**Comorbidities**	**HD**		**PD**		***p*** **Value**
	***n***	**%**	***n***	**%**	
**Diabetes**	23	38	8	40	0.895
**Arterial hypertension**	57	95	19	95	1.000
**Cardiovascular disease**	29	48	8	40	0.517
**Chronic coronary syndrome**	18	30	6	30	1.000
**Past myocardial infarction**	9	15	3	15	1.000
**Past stroke**	6	10	2	10	1.000
**Chronic heart failure**	11	18	3	15	0.734

BMI: body mass index; BSA: body surface area (BSA Dubois calculated according to formula BSA = 0.007184 × Height^0.725^ × Weight^0.425^); CRP: C-reactive protein; DBP: diastolic blood pressure; GFR, glomerular filtration rate; HGB: hemoglobin; HR: heart rate; SBP: systolic blood pressure; SD: standard deviation; PD: peritoneal dialysis; HD: hemodialysis. ^i^ urine collection in HD group have been done at the day off dialysis.

**Table 7:** Results of the clinical assessment of patients.

Scales	Class	HD		PD		*p* Value
		*N*	%	*n*	%	
**NYHA**	I	29	48	9	45	0.796
	II	15	25	7	35	0.386
	III + IV	16	27	4	20	0.773
**LUST**	1	35	58	11	55	0.794
	2	10	17	3	15	0.861
	3	10	17	5	25	0.408
	4	5	8	1	5	0.624
**Odema**	0	37	62	9	45	0.192
	1	9	15	4	20	0.599
	2	11	18	5	25	0.519
	3	3	5	2	10	0.424

PD: peritoneal dialysis; HD: hemodialysis; LUST: Lung Water by Ultrasound Guided Treatment in Hemodialysis Patients; NYHA: New York Heart Association; ns: not significant.

**Table 8:** Comparison of the number of patients in different hydration states.

Different Volume State Measurements		HD		PD		*p* Value
		*n*	%	*n*	%	
Clinical evaluation	Non-Hypervolemic	15	25	2	10	0.156
	Hypervolemia	45	75	18	90	0.156
LUS	No pulmonary congestion	30	50	9	45	0.327
	Mild-to-sever pulmonary congestion	30	50	11	55	0.368
IVCDi	Hypovolemia	1	2	1	5	0.385
	Euvolemia	29	48	8	42	0.635
	Hypervolemia	30	50	10	53	0.842
BCM-OH	Hypovolemia	0	0	1	5	0.082
	Euvolemia	29	49	8	40	0.478
	Hypervolemia	30	51	11	55	0.205

PD: peritoneal dialysis; HD: hemodialysis; LUS: Lung Ultrasound; BCM: body composition monitor; BCM-OH: overhydration according to BCM; IVCDi: inferior vena cava diameter index.

**Table 9:** Kappa test of Cohen for HD + PD patients.

	Clinical Evaluation	Edema	LUST	NYHA
LUS	0.10	0.35	0.31	0.00
IVCDi	0.21	0.49	0.24	0.04
BCM-OH	0.30	0.50	0.47	0.06

PD: peritoneal dialysis; HD: hemodialysis; BCM: body composition monitor; BCM-OH: over-hydration according to BCM; IVCDi: inferior vena cava diameter index; LUST: Lung Water by Ultrasound Guided Treatment in Hemodialysis Patients; NYHA: New York Heart Association; ns, not significant; LUS: Lung Ultrasound.

**Table 10:** Comparison of the results of select studies that objectify the clinical assessment of overhydration.

Parameters	HD		PD		*p* Value
	Median	min–max	Median	min–max	
**B-line score**	5.0	0.0–151.0	6.0	1.0–50.0	0.453
**BCM-OH (L)**	1.20	−0.80–11.3	1.65	−2.8–4.8	0.554
**TFC/h (1/kOhm × 1/m)**	19.42	13.27–40.12	18.07	11.14–24.93	0.139
**IVCDi (mm/m^2^)**	11.56	7.74–19.3	11.95	7.7–15.5	0.726
**IVCCi (%)**	30.49	5.26–53.02	29.00	12.64–44.24	0.497
**LVMI (g/m^2^)**	181.59	108.72–382.28	150.99	106.47–256.70	0.063
**NT-proBNP (pg/mL)**	5 269.5	229.8–218 848.0	2241.0	327.7–155 528.0	0.127

BCM: body composition monitor; BCM-OH: overhydration according to BCM; IVCCi: inferior vena cava collapsibility index; IVCDi: inferior vena cava diameter index; LVMI: left ventricular mass index; NT-proBNP: N-terminal pro B-type natriuretic peptide; TFC/h: thoracic fluid content indexed to height.
